# A case report of TAFRO syndrome successfully treated by immunosuppressive therapies with plasma exchange

**DOI:** 10.1007/s00277-018-3456-9

**Published:** 2018-07-27

**Authors:** Yusuke Meguri, Noboru Asada, Yukio Nakasako, Eisei Kondo, Yui Kambara, Akira Yamamoto, Taro Masunari, Nobuo Sezaki, Genyo Ikeda, Tomohiro Toji, Tadashi Yoshino, Toru Kiguchi

**Affiliations:** 1 Department of Hematology and Oncology, Chugoku Central Hospital, 148-13 Miyuki-Cho Kamiiwanari, Fukuyama, Hiroshima, 720-0001 Japan; 2 Department of Hematology and Oncology, Okayama University Hospital, Okayama, Japan; 3 Department of Nephrology, Chugoku Central Hospital, Fukuyama, Japan; 4 Department of General Medicine, Okayama University Hospital, Okayama, Japan; 5 Department of Respiratory Medicine, Chugoku Central Hospital, Fukuyama, Japan; 6 Department of Pathology, Chugoku Central Hospital, Fukuyama, Japan; 7 Department of Pathology, Dentistry, and Pharmaceutical Sciences, Okayama University Graduate School of Medicine, Okayama, Japan

Dear Editor,

TAFRO syndrome is an emerging clinical entity, presenting as systemic inflammatory disorder characterized by thrombocytopenia (T), anasarca (A), fever (F), reticulin fibrosis, renal failure (R), and organomegaly (O). Together, these clinical features have been described as TAFRO syndrome, which is considered to be the aggressive variant of multicentric Castleman’s disease (MCD) [1]. We report the first case for which plasma exchange (PE) was effective in treating severe clinical symptoms presenting as multi-organ failure (MOF).

A 52-year-old woman presented with fever and systemic edema. The blood examination showed thrombocytopenia, anemia, renal failure, and elevated inflammation markers (CRP, 25.3 mg/dl; interleukin [IL]-6, 250 pg/ml). Computed tomography revealed multiple lymphadenopathies. The histopathological findings of the mediastinal mass and the bone marrow biopsy showing reticulin fibrosis were compatible with TAFRO syndrome. Steroid pulse, cyclophosphamide, tocilizumab, and rituximab were given under mechanical ventilation for respiratory failure, and continuous hemodiafiltration (CHDF) was given for renal failure. However, clinical features, including hyper-bilirubinemia (T.Bil, 17 mg/dl), rapidly worsened. We started PE for MOF with liver dysfunction. After the initiation of PE, the jaundice quickly declined, renal function began to improve, and systemic edema lessened. Eventually, CHDF was discontinued. While the patient’s prognosis was complicated by the development of severe *Stenotorophomonous maltophilia* (*S. maltophilia*) pneumonia and bacterial sepsis, the features of TAFRO, including renal dysfunction and jaundice, began to improve. Consequently, she recovered from MOF and the severe infections, and was eventually discharged with complete recovery.

The primary purpose of treatment of TAFRO is to control systemic inflammation, as the aberrant increase of inflammatory cytokines, including IL-6 and vascular endothelial growth factor, have been suggested as the pathogenesis of TAFRO syndrome. In some case series of TAFRO syndrome [2], the majority of patients were treated with corticosteroids, and cyclosporine or tocilizumab were also reported to be effective [3]. Chemotherapies such as CHOP, rituximab, bortezomib, and thalidomide have also been used [4]. Intriguingly, one case achieved a remission of clinical symptoms for 6 months through only a debulking surgery of a large mediastinal mass [5]. Thus, both reduction and suppression of cytokine-producing cells seem to be important for treating TAFRO syndrome. In the current case, severe symptoms and bilirubinemia persisted after intensive treatment with immunosuppressive therapies. However, immediately after initiation of PE, severe clinical features were improved, suggesting that PE might be a useful therapeutic option in patients with TAFRO syndrome refractory to intensive immunosuppressive therapies.

**Fig. 1:**
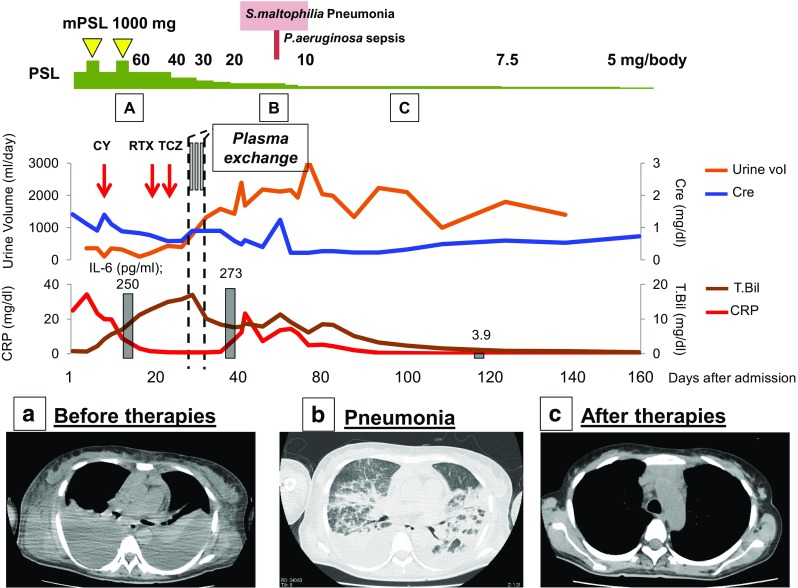
Clinical course with immunosuppressive therapies, manifestations and graphs of laboratory findings. CT-scan findings showed anasarca, pleural effusion, and mediastinal lymphadenopathy before therapies (**a**), when pneumonia was developed (**b**), and after therapies (**c**)

PE has been widely used to remove pathogenic substances, such as cytokines, antibodies, antigen-complexes, and bilirubin, from patients’ plasma. Although IL-6 is generally thought to have a strong correlation with the pathogenesis of MCD and TAFRO syndrome, the level of IL-6 in the serum of the present patient after PE remained high (273 pg/ml, Fig. 1). As the patient developed *S. maltophilia* pneumonia after PE, we assumed that the level of IL-6 had already re-ascended at that point due to the infection. Thus, we used a clinical finding (urine volume) and serum levels of creatinine or bilirubin as markers for monitoring the patient’s response to the therapy. While some studies reported that IL-8, tumor necrosis factor (TNF)-α, and IFN-γ could be removed by PE [6–8], IL-6 was not removed in some of these reports [8, 9]. Iwaki et al. reported that serum IFN-γ-induced protein 10 kDa (IP-10), a cytokine belonging to the CXC chemokine family, is specifically elevated in TAFRO syndrome, suggesting that IP-10 or other undefined cytokines might be involved in the pathogenesis of TAFRO syndrome [10]. Although these cytokines were studied as diagnostic tools, it has not been elucidated whether they directly reflect the treatment effect. Defining surrogate markers for monitoring disease status requires further investigation.

Our case suggests that a combination of the cytotoxic therapy and cytokine-removing therapy such as PE could be a therapeutic option for TAFRO syndrome with severe inflammation.
